# Recovery after stroke: not so proportional after all?

**DOI:** 10.1093/brain/awy302

**Published:** 2018-12-07

**Authors:** Thomas M H Hope, Karl Friston, Cathy J Price, Alex P Leff, Pia Rotshtein, Howard Bowman

**Affiliations:** 1 Wellcome Centre for Human Neuroimaging, University College London, UK; 2 Institute of Cognitive Neuroscience, University College London, UK; 3 Department of Brain Repair and Rehabilitation, Institute of Neurology, University College London, UK; 4 School of Psychology, University of Birmingham, UK; 5 School of Computing, University of Kent, UK

**Keywords:** proportional recovery, stroke, methods, statistics, outcomes

## Abstract

The proportional recovery rule asserts that most stroke survivors recover a fixed proportion of lost function. To the extent that this is true, recovery from stroke can be predicted accurately from baseline measures of acute post-stroke impairment alone. Reports that baseline scores explain more than 80%, and sometimes more than 90%, of the variance in the patients’ recoveries, are rapidly accumulating. Here, we show that these headline effect sizes are likely inflated. The key effects in this literature are typically expressed as, or reducible to, correlation coefficients between baseline scores and recovery (outcome scores minus baseline scores). Using formal analyses and simulations, we show that these correlations will be extreme when outcomes are significantly less variable than baselines, which they often will be in practice regardless of the real relationship between outcomes and baselines. We show that these effect sizes are likely to be over-optimistic in every empirical study that we found that reported enough information for us to make the judgement, and argue that the same is likely to be true in other studies as well. The implication is that recovery after stroke may not be as proportional as recent studies suggest.

## Teaser

The proportional recovery rule asserts that most stroke survivors recover a fixed proportion of lost function. Reports that the rule accurately predicts empirical recovery are rapidly accumulating. However, Hope *et al.* show that there is a fallacy at the heart of the rule that confounds many of these results.

## Introduction

Clinicians and researchers have long known stroke patients’ initial symptom severity is related to their longer term outcomes (Jongbloed, 1986). Recent studies have suggested that this relationship is stronger than previously thought: that most patients recover a fixed proportion of lost function. Studies supporting this ‘proportional recovery rule’ are rapidly accumulating (Stinear, 2017): in five studies since 2015 (Byblow *et al.*, 2015; Feng *et al.*, 2015; Winters *et al.*, 2015; Buch *et al.*, 2016; Stinear *et al.*, 2017), researchers used the Fugl-Meyer scale to assess patients’ upper limb motor impairment within 2 weeks of stroke onset (‘baselines’), and then again either 3 or 6 months post-stroke (‘outcomes’). The results were consistent with earlier observations (Prabhakaran *et al.*, 2008; Zarahn *et al.*, 2011) that most patients recovered ∼70% of lost function. Taken together, these studies report highly consistent recovery in over 500 patients, across different countries with different approaches to rehabilitation, regardless of the patients’ ages at stroke onset, stroke type, sex, or therapy dose (Stinear, 2017). And there is increasing evidence that the rule also captures recovery from post-stroke impairments of lower limb function (Smith *et al.*, 2017), attention (Marchi *et al.*, 2017; Winters *et al.*, 2017), and language (Lazar *et al.*, 2010; Marchi *et al.*, 2017), and may even apply generally across cognitive domains (Ramsey *et al.*, 2017). Even rats appear to recover proportionally after stroke (Jeffers *et al.*, 2018).

Strikingly, many of these studies report that the baseline scores predict 80–90%, or more, of the variance in empirical recovery. When predicting behavioural responses in humans, these effect sizes are unprecedented. Recently, Winters and colleagues (2015) reported that recovery predicted from baseline scores explained 94% of the variance in the empirical recovery of 146 stroke patients. Like many related reports (Stinear, 2017), this study also reported a group of (*n* = 65) ‘non-fitters’, who did not make the predicted recovery. But if non-fitters can be distinguished at the acute stage, as this and other studies suggest (Stinear, 2017), the implication is that we can predict most patients’ recovery near-perfectly, given baseline scores alone. Stroke researchers are used to thinking of recovery as a complex, multi-factorial process (Nelson *et al.*, 2016). If the proportional recovery rule is as powerful as it seems, post-stroke recovery is simpler and more consistent than previously thought.

In what follows, we argue that the empirical support for proportional recovery is weaker than it seems. These results are typically expressed as, or reducible to, correlations between baselines and recovery (outcomes minus baselines). These analyses pose well known challenges that have been discussed by statisticians for decades (Lord, 1956; Oldham, 1962; Cronbach and Furby, 1970; Hayes, 1988; Tu *et al.*, 2005). Much of this discussion is focused on problems induced by measurement noise, and measurement noise was also the focus of the only prior application of that discussion to the proportional recovery rule (Krakauer and Marshall, 2015). Here, we argue that empirical studies of proportional recovery after stroke are likely confounded entirely regardless of measurement noise.

Our argument is that: (i) correlations between baselines and recovery are spurious when they are stronger than correlations between baselines and outcomes; (ii) this is likely when outcomes are less variable than baselines; which (iii) will often happen in practice, whether or not recovery is proportional. This argument follows from a formal analysis of correlations between baselines and recovery, which we introduce below and illustrate with examples. Armed with that analysis, we then re-examine the empirical support for the proportional recovery rule.

## The relationships between baselines, outcomes, and recovery

For the sake of brevity, we define ‘baselines’ = X, ‘outcomes’ = Y, and ‘change’ (recovery) = Δ: i.e. Y − X. The ‘correlation between baselines and outcomes’ is r(X,Y), and the ‘correlation between baselines and change’ is r(X,Δ). Finally, we define the ‘variability ratio’ as the ratio of the standard deviation (σ) of Y to the standard deviation of X: σ_Y_/σ_X_.

X and Y are construed as lists of scores, with each entry being the performance of a single patient at the specified time point. We assume that higher scores imply better performance, so r(X,Δ) will be negative if recovery is proportional (to lost function). One can equally substitute ‘lost function’ (e.g. maximum score minus actual score), for ‘baseline score’, but while this makes r(X,Δ) positive if recovery is proportional, it is otherwise equivalent.

### Strong correlations imply the potential for accurate predictions

**Figure 1:**
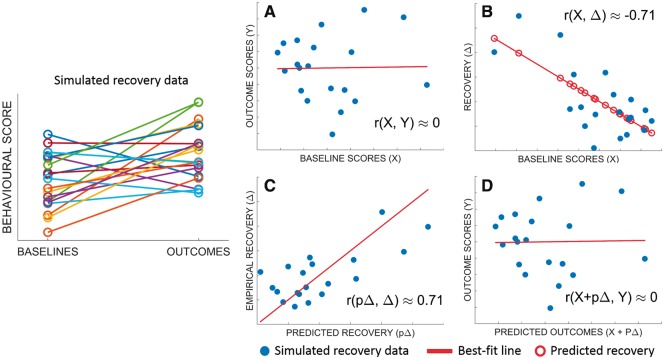
**A canonical example of spurious r(X, Δ).** Baselines scores are uncorrelated with outcomes (**A**), but baseline scores appear to be strongly correlated with recovery (**B**). That correlation can be used to derive predicted recovery, which is strongly correlated with empirical recovery (**C**), but predicted outcomes, derived from that predicted recovery, are still uncorrelated with empirical outcomes (**D**).

Strong correlations between any two variables typically imply that we can use either variable to predict the other. Out-of-sample predictions should tend toward the least-squares line defined by the original (in-sample) correlation. Some empirical studies use this logic to derive ‘predicted recovery’ (pΔ) from the least-squares line for r(X,Δ), reporting r(pΔ,Δ) instead of r(X,Δ) (Winters *et al.*, 2015; Marchi *et al.*, 2017). Since the magnitudes of r(X,Δ) and r(pΔ,Δ) are the same by definition (see Fig. 1 and Supplementary material, proposition 8 in Appendix A), the preference for either expression over the other is arguably cosmetic.

Nevertheless, the correlation between predicted and empirical data is a common measure of predictive accuracy: the stronger the correlation, the better the predictions. Very strong correlations are unusual when predicting behavioural performance in humans—both because behaviour itself is complex, and because of measurement noise in behavioural assessment. Once r(pΔ,Δ) > ∼0.95, for example (Winters *et al.*, 2015), this prognostic problem has seemingly been ‘solved’ more accurately than many might have thought possible.

### r(X,Δ) is spurious when (non-trivially) stronger than r(X,Y)

Recovery is precisely the difference between baselines and outcomes. When r(X,Δ) is strong, implying that we can predict recovery accurately given baselines, it is tempting to assume that we can also predict outcomes equally accurately, by simply adding predicted recovery to baselines. More formally, the assumption is that r(X + pΔ,Y) ≈ r(pΔ,Δ). This assumption is wrong.

In fact, r(X + pΔ,Y) ≈ r(X,Y) (see Fig. 1 and Supplementary material, proposition 8 in Appendix A). When recovery is predicted from baselines, the correlation between ‘baselines plus predicted recovery’ and outcomes, is never stronger than the correlation between baselines and outcomes. When r(X,Δ) is (substantially) stronger than r(X,Y), r(X,Δ) is ‘spurious’, because it encourages an over-optimistic impression of how predictable outcomes are, given baselines.

### The canonical example of spurious r(X,Δ)

The canonical example of spurious r(X,Δ) is when X and Y are independent random variables with the same variance: σ_Y_/σ_X_ ≈ 1 and r(X,Y) ≈ 0, but r(X,Δ) ≈ −0.71 (Oldham, 1962). This r(X,Δ) suggests that we can predict recovery relatively well, but we cannot use ‘predicted recovery’ to predict outcomes equally well (Fig. 1).

Krakauer and Marshall (2015) recently argued that this scenario has little relevance to (most) empirical studies of recovery after stroke. This is because: (i) spurious r(X,Δ) only emerge here when r(X,Y) is weak; and (ii) empirical r(X,Y) are usually strong, because X and Y are dependent, repeated measurements from the same patients. If spurious r(X,Δ) only or mainly emerged when σ_Y_/σ_X_ ≈ 1 and r(X,Y) ≈ 0, they might indeed be irrelevant in practice. Unfortunately, spurious r(X,Δ) also emerge in another scenario, which is very common in studies of recovery after stroke.

### Spurious r(X,Δ) are likely when σ_Y_/σ_X_ is small

For any X and Y, it can be shown that:

(1) $$r\left(X,\Delta \right)=\frac{\sigma_{Y}.r\left(X,Y\right)-\sigma_{X}}{\sqrt{\sigma_{Y}^{2}+\sigma_{X}^{2}-2.\sigma_{X}.\sigma_{Y}.r\left(X,Y\right)}}$$

**Figure 2:**
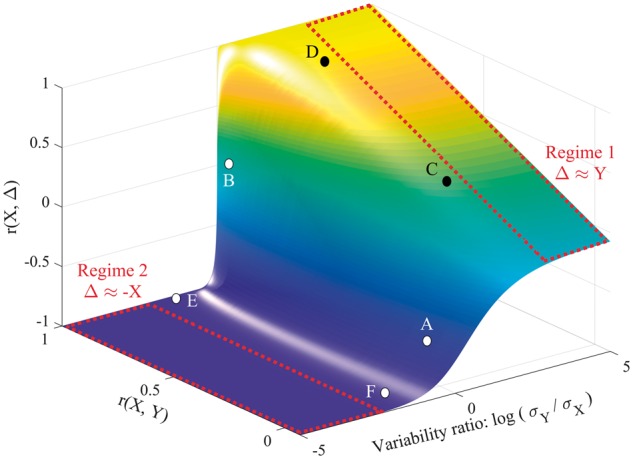
**The relationship between r(X,Y), r(X,Δ) and σ_Y_/σ_X_.** Note that the *x*-axis is log-transformed to ensure symmetry around 1; when X and Y are equally variable, log(σ_Y_/σ_X_) = 0. Supplementary material, proposition 7 in Appendix A, provides a justification for unambiguously using a ratio of standard deviations in this figure, rather than σ_Y_ and σ_X_ as separate axes. The two major regimes of Equation 1 are also marked in red. In Regime 1, Y is more variable than X, so contributes more variance to Δ, and r(X,Δ) ≈ r(X,Y). In Regime 2, X is more variable than Y, so X contributes more variance to Δ, and r(X,Δ) ≈ r(X,−X) (i.e. −1). The transition between the two regimes, when the variability ratio is not dramatically skewed either way, also allows for spurious r(X,Δ). For the purposes of illustration, the figure also highlights six points of interest on the surface, marked A–F; examples of simulated recovery data corresponding to these points are provided in Fig. 3.

**Figure 3:**
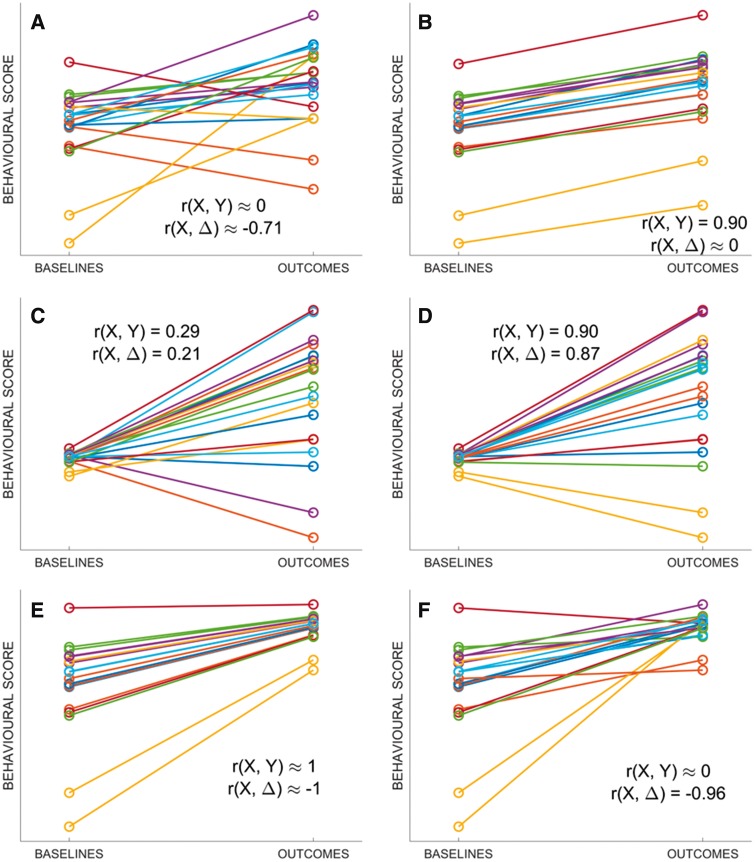
**Exemplar points on the surface in** **Fig. 2** **. **Simulated recovery data, corresponding to the points A–F marked on the surface in Fig. 1. (**A**) Baselines and outcomes are entirely independent [r(X,Y) = 0], yet r(X,Δ) is relatively strong; this is the canonical example of mathematical coupling, first introduced by Oldham (1962). (**B**) Recovery is constant with minimal noise, so baselines and outcomes are equally variable (σ_Y_/σ_X_ ≈ 1) and recovery is unrelated to baseline scores (r(X, Δ) ≈ 0). (**C** and **D**) Outcomes are more variable than baselines (σ_Y_/σ_X_ ≈ 5), and r(X,Δ) converges to r(X,Y). (**E**) Recovery is 70% of lost function, so outcomes are less variable than baselines (σ_Y_/σ_X_ ≈ 0.3); even with shuffled outcomes data (**F**) baselines and recovery still appear to be strongly correlated.

A formal proof of Equation 1 is provided in the Supplementary material, Appendix A [proposition 4 and theorem 1; also see (Oldham, 1962)]; its consequence is that r(X,Δ) is a function of r(X,Y) and σ_Y_/σ_X_. To illustrate that function, we performed a series of simulations (Supplementary material, Appendix B) in which r(X,Y) and σ_Y_/σ_X_ were varied independently. Figure 2 illustrates the results: a surface relating r(X,Δ) to r(X,Y) and σ_Y_/σ_X_. Figure 3 shows example recovery data at six points of interest on that surface.

Point A corresponds to the canonical example of spurious r(X,Δ), introduced in the last section: i.e. σ_Y_/σ_X_ ≈ 1 and r(X,Y) ≈ 0, but r(X,Δ) ≈ −0.71 (Fig. 3A). At Point B, σ_Y_/σ_X_ ≈ 1 and r(X,Y) is strong, so recovery is approximately constant (Fig. 3B) and r(X,Δ) ≈ 0, consistent with the view that strong r(X,Y) curtail spurious r(X,Δ) (Krakauer and Marshall, 2015). However, the situation is more complex when σ_Y_/σ_X_ is more skewed.

When σ_Y_/σ_X_ is large, Y contributes more variance to Y − X (Δ), and r(X,Δ) ≈ r(X,Y); this is Regime 1. Points C and D illustrate the convergence (Fig. 3C and D). By contrast, when σ_Y_/σ_X_ is small, X contributes more variance to Y − X, and r(X,Δ) ≈ r(X, −X): i.e. −1 (Supplementary material, Appendix A, theorem 2); this is Regime 2, where the confound emerges. Point E, near Regime 2, corresponds to data in which all patients recover proportionally (Δ = 70% of lost function; Fig. 2E). Here, σ_Y_/σ_X_ is already small enough (0.3) to be dangerous: after randomly shuffling Y, r(X,Y) ≈ 0, but r(X,Δ) is almost unaffected (Point F, and Fig. 3F). In other words, if even the proportional recovery rule is approximately right, empirical data may enter territory, on the surface in Fig. 2, where over-optimistic r(X,Δ) are likely.

### σ_Y_/σ_X_ may be small, whether or not recovery is proportional

Proportional recovery implies small σ_Y_/σ_X_, but small σ_Y_/σ_X_ does not imply proportional recovery; for example, constant recovery with ceiling effects will produce the same result. To illustrate this, we ran 1000 simulations in which: (i) 1000 baseline scores are drawn randomly with uniform probability from the range 0–65 (i.e. impaired on the 66-point Fugl-Meyer upper-extremity scale); (ii) outcome scores were calculated as the baseline scores plus half the scale’s range (33); and (iii) outcome scores greater than 66 were set to 66 (i.e. a hard ceiling). Mean r(X,Y) and r(X,Δ) were calculated both before and after shuffling the outcomes data for each simulation. After shuffling, r(X,Y) ≈ 0 and r(X,Δ) = −0.88: ceiling effects make σ_Y_/σ_X_ small enough to encourage spurious r(X,Δ). And just as importantly, before shuffling, r(X,Y) = 0.89 and r(X,Δ) = −0.90: even when r(X,Δ) is not spurious [because r(X,Y) is similarly strong], we cannot conclude that recovery is really proportional.

## Re-examining the empirical literature on proportional recovery

**Figure 4:**
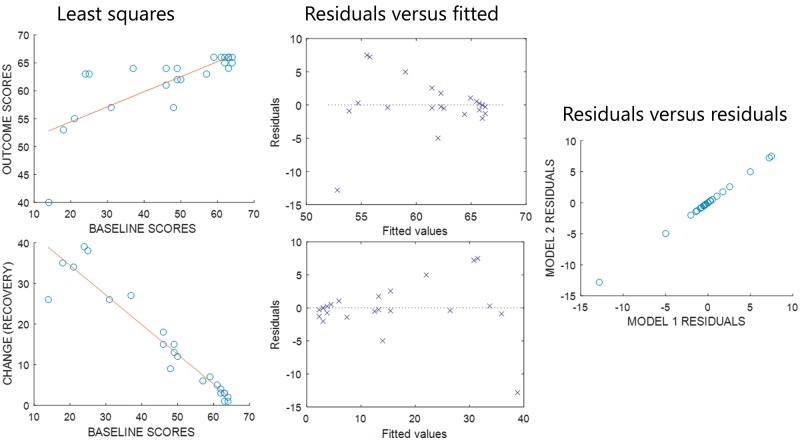
**r(X,Y) and r(X,Δ) have the same residuals.** *Left*: Least squares linear fits for analyses relating baselines to (*top*) outcomes and (*bottom*) recovery, using the fitters’ data reported by Zarahn *et al.* (2011). *Middle*: Plots of residuals relative to each least squares line, against the fitted values in each case. *Right*: A scatter plot of the residuals from the model relating baselines to change, against the residuals from the model relating baselines to outcomes: the two sets of residuals are the same.

The relationships between r(X,Y), r(X,Δ) and σ_Y_/σ_X_ merit a re-examination of the empirical support for the proportional recovery rule. In the only study we found, which reports individuals’ behavioural data, Zarahn and colleagues (2011) consider 30 patients’ recoveries from hemiparesis after stroke. Across the whole sample, r(X,Y) = 0.80 and r(X,Δ) = −0.49; after removing seven non-fitters: r(X,Y) = 0.75 and r(X,Δ) = −0.95. Removing the non-fitters increases the apparent predictability of recovery but reduces the predictability of outcomes (and reduces σ_Y_/σ_X_ from 0.88 to 0.36). Notably, the residuals for both correlations are identical (Fig. 4), and in fact this is always true (Supplementary material, proposition 9 in Appendix A,). r(X,Δ) has the same errors as r(X,Y), but a larger effect size: r(X,Δ) is over-optimistic.

We can also use Equation 1 to reinterpret studies that do not report individual patient data. One example is the first study to report proportional recovery from aphasia after stroke (Lazar *et al.*, 2010). Here, r(X,Δ) ≈ −0.9 and σ_Y_/σ_X_ ≈ 0.48; Equation 1 implies that r(X,Y) was either ∼0.78 or zero. Similarly, in the recent study of proportional recovery in rats (Jeffers *et al.*, 2018), σ_Y_/σ_X_ ≈ 0.8, and r(X,Δ) ≈ −0.71; by Equation 1 r(X,Y) was either much stronger (>0.95) or considerably weaker (∼0.29) than r(X,Δ). In both cases, r(X,Δ) tells us less than expected about how predictable outcomes really were, given baselines.

Several recent studies report interquartile ranges, rather than standard deviations, for their fitter patients’ baselines and outcomes. Accepting some room for error, we can also estimate σ_Y_/σ_X_ from those interquartile ranges. In one case (Winters *et al.*, 2015), r(X,Δ) = −0.97 and σ_Y_/σ_X_ = 0.158, while in another (Veerbeek *et al.*, 2018), σ_Y_/σ_X_ = 0.438 and r(X,Δ) ≈ −0.88. In both cases, Equation 1 implies that r(X,Δ) would be at least as strong as that reported, regardless of r(X,Y): these reported r(X,Δ) do not tell us how predictable outcomes actually were, given baseline scores.

Many studies in this literature only relate baselines to recovery through multivariable models (Buch *et al.*, 2016; Marchi *et al.*, 2017; Winters *et al.*, 2017); in these studies, we cannot demonstrate confounds directly with Equation 1. Nevertheless, these studies are also probably confounded, because any inflation in one variable’s effect size will inflate the multivariable model’s effect size as well. As discussed in the previous section, empirical studies of recovery after stroke should tend to encourage small σ_Y_/σ_X_, whether or not recovery is proportional. Consequently, the null hypothesis will rarely be that r(X,Δ) ≈ 0. For example, in the only multivariable modelling study, which reports IQRs for its fitter-patients’ baselines and outcomes (Stinear *et al.*, 2017), σ_Y_/σ_X_ ≈ 0.48, which implies that the weakest r(X,Δ) was −0.88, for any positive value of r(X,Y).

Finally, while r(X,Δ) can be misleading if it is extreme relative to r(X,Y), the reverse is also true. One study in this literature, which uses outcomes as the dependent variable rather than recovery (Feng *et al.*, 2015), reports that r(X,Y) ≈ 0.8 and σ_Y_/σ_X_ = 1.2 in their ‘combined’ group of 76 patients. By Equation 1, r(X,Δ) = −0.05: i.e. recovery was uncorrelated with baseline scores. These authors only reported proportional recovery in a subsample of their patients (but not the information we need to re-examine that claim), but their full sample seems better described by constant recovery (as in Fig. 3B).

## Discussion

The proportional recovery rule is striking because it implies that recovery is simple and consistent across patients (non-fitters notwithstanding), and because that implication appears to be justified by strong empirical results (Stinear, 2017). We contend that the empirical support for the rule is weaker than it seems.

In summary, our argument is that r(X,Δ) is spurious when stronger than r(X,Y), and that the conditions that encourage spurious r(X,Δ) will be common in empirical studies of recovery after stroke, whether or not recovery is really proportional. Many empirical r(X,Δ) in this literature appear to be spurious in this sense. And in any case, strong r(X,Δ) are insufficient evidence for proportional recovery even if they are not spurious [because r(X,Y) is similarly strong].

The only previous discussion of the risk of spurious r(X,Δ), in analyses of recovery after stroke (Krakauer and Marshall, 2015), concluded that this risk is small provided the tools used to measure post-stroke impairment are reliable: i.e. so long as measurement noise is minimal. Crucially, our analysis applies entirely regardless of measurement noise. We contend that the risk of spurious r(X,Δ) is significant, if there are ceiling effects on the scale used to measure post-stroke impairment, and if most patients improve between baseline and subsequent assessments. These criteria will usually be met in practice, because every practical measurement of post-stroke impairment employs a finite scale, and because non-fitters, who do not make the predicted recovery, are removed prior to calculating r(X,Δ).

We are not suggesting that there is anything wrong with the practice of distinguishing fitters from non-fitters. Indeed, our results prove that this work may be valid regardless of our other concerns. Non-fitters do not recover as predicted; by definition, they contribute the largest, negative residuals to r(X,Δ). Since the residuals for r(X,Y) and r(X,Δ) are identical (Fig. 4 and Supplementary material, proposition 9 in Appendix A), the same patients will be placed in the same subgroups regardless of which correlation is used, and biomarkers that distinguish those subgroups at the acute stage [i.e. avoid the circularity of relying on observed recovery (Stinear, 2017)], will be equally accurate regardless of our other concerns. However, extreme r(X,Δ) for patients classified as fitters, will naturally encourage the assumption that those fitters’ outcomes are largely determined by initial symptom severity. If this assumption is true, therapeutic interventions must be largely ineffective (or at least redundant) for these patients. Our analysis suggests that this assumption is wrong.

Nevertheless, we are not claiming that the proportional recovery rule is wrong. Our analysis suggests that empirical studies to date do not demonstrate that the rule holds, or how well, but we could only confirm that r(X,Δ) was over-optimistic in one study, which reported individual patient data. And while we have also shown that extreme r(X,Δ) and r(X,Y) can result from non-proportional (constant) recovery, this is simply one plausible alternative hypothesis about how patients recover.

Quite how to interpret empirical recovery with confidence in this domain remains an open question: we have articulated a problem here, hoping that recognition of the problem will motivate work to solve it. But we can make some recommendations for future studies in the field.

First, these studies should report r(X,Δ), r(X,Y), and σ_Y_/σ_X_, for those patients deemed to recover proportionally. Despite our concerns about r(X,Δ), we do learn something when r(X,Y) is strong, but r(X,Δ) is weak, as in Feng and colleagues’ (2015) results discussed above, which appeared to be better explained by constant recovery than by proportional recovery.

Second, future studies should consider explicitly testing the hypothesis that recovery depends on baseline scores (Oldham, 1962; Hayes, 1988; Tu *et al.*, 2005; Tu and Gilthorpe, 2007; Chiolero *et al.*, 2013). These tests sensibly acknowledge that the null hypothesis is rarely r(X,Δ) ≈ 0 in these analyses. However, they do not address the proper measurement and interpretation of effect sizes, which is our primary concern here; somewhat paradoxically, this means that they may be less useful in larger samples than in smaller samples (Friston, 2012; Lorca-Puls *et al.*, 2018).

Those hypothesis tests will also all be confounded by ceiling effects. We recommend that future studies should measure the impact of such effects, perhaps by reporting the shapes of the distributions of X and Y (greater asymmetry implying more prominent ceiling effects). Future studies should also attempt to minimize ceiling effects. One approach might be to remove patients whose outcomes are at ceiling: though certainly inefficient, this does at least remove the spurious r(X,Δ) in our simulations of constant recovery (see above). However, it may be difficult to determine which patients to remove in practice; the Fugl-Meyer scale, for example, imposes item-level ceiling effects, which could distort σ_Y_/σ_X_ well below the maximum score. A better, though also more complex alternative, may be to use assessment tools expressly designed to minimize ceiling effects, or to add such tools to those currently in use.

More generally, we may need to replace correlations with alternative methods, which can provide less ambiguous evidence for the proportional recovery rule. One principled alternative might use Bayesian model comparison to adjudicate between different forward or generative models of the data at hand: i.e. using the empirical data to quantify evidence for or against competing hypotheses about the nature of recovery, which may or may not be conserved across patients. We hope that this paper will encourage work to develop such methods, delivering better evidence for (or against) the proportional recovery rule.

## Funding

This study was supported by the Medical Research Council (MR/M023672/1), Wellcome (091593/Z/10/Z and 205103/Z/16/Z), and the Stroke Association (TSA PDF 2017/02; TSA 2014/02).

## Competing interests

The authors report no competing interests.

## Supplementary Material

Supplementary Appendix
