# Supplementary material for: Recovery after stroke: not so proportional after all?
Source: Brain. 2018 Dec 7;142(1):15–22. doi: 10.1093/brain/awy302 (PMC6308308; doi:10.1093/brain/awy302)
Supplement: Supplementary Appendix [file awy302_supplementary_appendix.pdf]

Supplementary Appendix A: formal relationships between the correlations

We present a simple, general and self-contained formulation of the proportional recovery concept. We have derived all of the key results from first principles, while acknowledging previous presentations of these results when they can be found in the literature.

We assume two variables X' and Y' corresponding to performance at initial test (X') and at second test (Y'). These will be represented as column vectors, with each entry being the performance of a single patient and vector lengths being $N \in \mathbb{N}$. Performance improves as numbers get bigger, up to a maximum, denoted Max, which corresponds to no discernible deficit. Severity is measured as difference from maximum, i.e. $Max - X'$.

The two variables (X' and Y') could be specialised to more detailed formulations: e.g., true score theory or with an explicit modelling of measurement or state error. However, this would not impact any of the derivations or inferences that follow. Indeed, the results that we present would hold even in the complete absence of measurement noise, which has been considered the main concern for the validity of quantifications of proportional recovery.

Demeaning

Without loss of generality, we work with demeaned variables. That is, where over-lining denotes mean, we define new variables as,

$$X = X' - \overline{X}'$$

$$Y = Y' - \overline{Y}'$$

This also means that recovery, i.e. $Y - X$, will be demeaned, since, using proposition 1, the following holds.

$$Y - X = (Y' - \overline{Y'}) - (X' - \overline{X'}) = (Y' - X') - (\overline{Y'} - \overline{X'}) = (Y' - X') - \overline{(Y' - X')}$$

Proposition 1

Let V and W be vectors of the same length, denoted N. Then, the following holds,

$$\bar{V} + \bar{W} = \overline{(V + W)}$$

with $\bar{V} - \bar{W} = \overline{(V - W)}$ as a trivial consequence.

Proof

By distributivity of multiplication through addition and associativity of addition, the following holds.

$$\bar{V} + \bar{W} = \left(\frac{1}{N}\sum_{i=1}^{N} V_i\right) + \left(\frac{1}{N}\sum_{i=1}^{N} W_i\right) = \frac{1}{N}\left(\sum_{i=1}^{N} V_i + \sum_{i=1}^{N} W_i\right) = \frac{1}{N}\left(\sum_{i=1}^{N}(V_i + W_i)\right) = \overline{(V + W)}$$

QED

Correlations

There are two basic correlations we are interested in, (1) the correlation between initial performance and performance at second test, i.e. $r(X, Y)$, and (2) the correlation between initial performance and recovery, i.e. $r(X, Y - X) = r(X, \Delta)$. The latter of these is the key relationship, and

we would expect this to be a negative correlation; that is, as initial performance is smaller (i.e. further from Max), the larger is recovery. (One could also formulate the correlation as $r((Max - X), Y - X)$, which would flip the correlation to positive, but the two approaches are equivalent).

Our main correlations are defined as follows,

$$r(X, Y) = \frac{\sum_{i=1}^{N} X_i \,.\, Y_i}{(N-1)\,.\,\sigma_X\,.\,\sigma_Y}$$

$$r(X, (Y - X)) = \frac{\sum_{i=1}^{N} (X_i \,.\, (Y_i - X_i))}{(N-1)\,.\,\sigma_X\,.\,\sigma_{(Y-X)}}$$

Standard Deviation of a Difference

We need a straightforward result on the standard deviation of a difference.

Proposition 2

$$\sigma_{(A-B)} = \sqrt{\sigma_A^2 + \sigma_B^2 - 2\,.\,cov(A, B)}$$

Proof

The result is a direct consequence of the following standard result from probability theory, e.g. see Ross, S. M. (2014). *Introduction to probability and statistics for engineers and scientists*. Academic Press.,

$$\sigma_{(A-B)}^2 = \sigma_A^2 + \sigma_B^2 - 2\,.\,cov(A, B)$$

Key Results

The following proposition enables us to express the key correlation, $r(X, (Y - X))$, in terms of covariance of its constituent variables.

Proposition 3

$$r(X, (Y - X)) = \frac{cov(X, Y) - cov(X, X)}{\sigma_X\,.\,\sqrt{\sigma_Y^2 + \sigma_X^2 - 2\,.\,cov(X, Y)}}$$

Proof

Using distributivity of multiplication through addition, associativity of addition, the definition of covariance and proposition 2, we can reason as follows.

$$r(X, (Y - X)) = \frac{\sum_{i=1}^{N} (X_i \,.\, (Y_i - X_i))}{(N-1)\,.\,\sigma_X\,.\,\sigma_{(Y-X)}} = \frac{\sum_{i=1}^{N} (X_i\, Y_i - X_i\, X_i)}{(N-1)\,.\,\sigma_X\,.\,\sigma_{(Y-X)}} = \frac{\sum_{i=1}^{N} (X_i\, Y_i) - \sum_{i=1}^{N} (X_i\, X_i)}{(N-1)\,.\,\sigma_X\,.\,\sigma_{(Y-X)}}$$

$$= \frac{cov(X, Y) - cov(X, X)}{\sigma_X \cdot \sigma_{(Y-X)}} = \frac{cov(X, Y) - cov(X, X)}{\sigma_X\,.\,\sqrt{\sigma_Y^2 + \sigma_X^2 - 2\,.\,cov(X, Y)}}$$

QED

It is straightforward to adapt proposition 3 to be fully in terms of correlations.

Proposition 4

$$r(X,(Y-X)) = \frac{\sigma_Y \,.\, r(X,Y) - \sigma_X \,.\, r(X,X)}{\sqrt{\sigma_Y^2 + \sigma_X^2 - 2\,.\,\sigma_X\,.\,\sigma_Y . r(X,Y)}}$$

Proof

Straightforward from proposition 3 and definition of correlations, which gives the relationship $cov(A,B) = \sigma_A\,.\,\sigma_B\,.\,r(A,B)$. QED

Scale Invariance

The next set of propositions justifies working with a standardised X variable.

Lemma 1

$$\forall c \in \mathbb{R} \cdot |c|.\sigma_A = \sigma_{(c.A)}$$

Proof

Using distributivity of a multiplicative constant through averaging, $\sqrt{d^2} = |d|$ and distributivity of square root through multiplication, we can reason as follows.

$$\sigma_{(c.A)} = \sqrt{\frac{\sum_{i=1}^{N} (c.A_i - \overline{c.A})^2}{N-1}} = \sqrt{\frac{\sum_{i=1}^{N} (c.A_i - c.\bar{A})^2}{N-1}} = |c|\,.\sqrt{\frac{\sum_{i=1}^{N} (A_i - \bar{A})^2}{N-1}} = |c|.\sigma_A$$

QED

Proposition 5 (Invariance to scaling)

The absolute magnitude of a correlation is not changed by scaling either variable by a constant, i.e.

$$\forall c \in \mathbb{R} \cdot r(A,B) = sign(c).r(c.A,B) = sign(c).r(A,c.B)$$

where $sign(d) = if\ (d < 0)\ then\ -1\ else\ +1$.

Proof

For any $c \in \mathbb{R}$, using distributivity of multiplication through mean and addition, and lemma 1, the following holds,

$$r(c.A,B) = \frac{\sum_{i=1}^{N} (c.A_i - \overline{c.A})(B_i - \bar{B})}{(N-1)\,.\,\sigma_{(c.A)}\,\sigma_B} = \frac{\sum_{i=1}^{N} (c.A_i - c.\bar{A})(B_i - \bar{B})}{(N-1)\,.\,\sigma_{(c.A)}\,\sigma_B}$$

$$= \frac{c\,.\sum_{i=1}^{N} (A_i - \bar{A})(B_i - \bar{B})}{(N-1)\,.\,|c|\,.\,\sigma_A\,.\,\sigma_B} = \frac{sign(c).\sum_{i=1}^{N} (A_i - \bar{A})(B_i - \bar{B})}{(N-1)\,.\,\sigma_A\,.\,\sigma_B} = sign(c).r(A,B)$$

Then, one can multiply both sides by $sign(c)$ to obtain $r(A,B) = sign(c).r(c.A,B)$. Additionally, as correlations are symmetric, $sign(c).r(c.B,A) = sign(c).r(A,c.B)$, and the full result follows.

QED

Corollary 1

$$\forall c \in \mathbb{R} \cdot r(A,B) = r(c.A,c.B)$$

Proof

Follows from twice applying proposition 5, and that $sign(c)^2 = +1$.   QED

Proposition 6

$$\forall c \in \mathbb{R} \cdot r(X,(Y-X)) = r(c.X,(c.Y-c.X))$$

Proof

We can use distributivity of multiplication through subtraction and corollary 1 to give us the following.

$$r\big(c.X,(c.Y-c.X)\big) = r\big(c.X,c.(Y-X)\big) = r(X,(Y-X))$$

QED

It follows from proposition 6 that we can work with a standardised X variable, since,

$$r(X/\sigma_X,(Y/\sigma_X - X/\sigma_X)) = r(X,(Y-X))$$

Proposition 7 (Sufficiency of variability ratio)

Assume two pairs of variables: X_1, Y_1 and X_2, Y_2, such that, $r(X_1,Y_1) = r(X_2,Y_2)$, then,

$$\frac{\sigma_{Y_1}}{\sigma_{X_1}} = \frac{\sigma_{Y_2}}{\sigma_{X_2}} \implies r\big(X_1,(Y_1-X_1)\big) = r\big(X_2,(Y_2-X_2)\big)$$

Proof

The proof has two parts.

1) We consider the implications of equality of ratio of standard deviations. Firstly, we note that,

$$\frac{\sigma_{Y_1}}{\sigma_{X_1}} = \frac{\sigma_{Y_2}}{\sigma_{X_2}} \iff \frac{\sigma_{X_2}}{\sigma_{X_1}} = \frac{\sigma_{Y_2}}{\sigma_{Y_1}} \qquad (eqn\ ratios)$$

Secondly, using eqn ratios, we can argue as follows,

$$\frac{\sigma_{Y_1}}{\sigma_{X_1}} = \frac{\sigma_{Y_2}}{\sigma_{X_2}} \iff \left(\sigma_{Y_2} = \frac{\sigma_{X_2}}{\sigma_{X_1}}\,\sigma_{Y_1} \wedge \sigma_{X_2} = \frac{\sigma_{Y_2}}{\sigma_{Y_1}}\,\sigma_{X_1}\right) \iff \left(\sigma_{Y_2} = \frac{\sigma_{X_2}}{\sigma_{X_1}}\,\sigma_{Y_1} \wedge \sigma_{X_2} = \frac{\sigma_{X_2}}{\sigma_{X_1}}\,\sigma_{X_1}\right)$$

$$\implies \big(\exists d \in \mathbb{R} \cdot \sigma_{Y_2} = d.\sigma_{Y_1} \wedge \sigma_{X_2} = d.\sigma_{X_1}\big)$$

2) Using 4, the fact that $r(X_1,Y_1) = r(X_2,Y_2)$, the property just derived in part 1), with $d = \frac{\sigma_{X_2}}{\sigma_{X_1}}$ and rules of square roots, we can reason as follows,

$$r\big(X_2,(Y_2-X_2)\big) = \frac{\sigma_{Y_2}.r(X_2,Y_2) - \sigma_{X_2}.r(X_2,X_2)}{\sqrt{\sigma_{Y_2}^2 + \sigma_{X_2}^2 - 2.\sigma_{X_2}.\sigma_{Y_2}.r(X_2,Y_2)}} = \frac{\sigma_{Y_2}.r(X_1,Y_1) - \sigma_{X_2}.r(X_1,X_1)}{\sqrt{\sigma_{Y_2}^2 + \sigma_{X_2}^2 - 2.\sigma_{X_2}.\sigma_{Y_2}.r(X_1,Y_1)}}$$

$$= \frac{d.\sigma_{Y_1}.r(X_1,Y_1) - d.\sigma_{X_1}.r(X_1,X_1)}{\sqrt{d^2.\sigma_{Y_1}^2 + d^2.\sigma_{X_1}^2 - 2.d.\sigma_{X_1}.d.\sigma_{Y_1}.r(X_1,Y_1)}} = \frac{d.(\sigma_{Y_1}.r(X_1,Y_1) - \sigma_{X_1}.r(X_1,X_1))}{d.\sqrt{\sigma_{Y_1}^2 + \sigma_{X_1}^2 - 2.\sigma_{X_1}.\sigma_{Y_1}.r(X_1,Y_1)}}$$

$$= r\big(X_1,(Y_1-X_1)\big).$$

QED

Proposition 8

If $\Delta = Y - X$ and $p\Delta = X.\beta$, where $\beta \in \mathbb{R}$, then,

1) $r(p\Delta, \Delta) = sign(\beta).r(X, \Delta)$; and
2) $r(X + p\Delta, \mathrm{Y}) = sign(1 + \beta).r(X, \mathrm{Y})$.

Proof

Both results are easy consequences of proposition 5.

1) $r(p\Delta, \Delta) = r(X.\beta, \Delta) = sign(\beta).r(X, \Delta)$.

2) $r(X + p\Delta, \mathrm{Y}) = r((X + (X.\beta)), \mathrm{Y}) = r((X.(1 + \beta)), \mathrm{Y}) = sign(1 + \beta).r(X, \mathrm{Y}) = r(X, \mathrm{Y})$.

QED

Main Findings

Theorem 1:

Since X will be standardised, we can adapt the finding in proposition 4, to give us the key relationship we need,

$$r\big(X, (Y - X)\big) = \frac{\sigma_Y \,.r(X, Y) - \sigma_X}{\sqrt{\sigma_Y^2 + 1 - 2\,.\sigma_Y.r(X, Y)}} \qquad (\text{eqn } Imprint)$$

Note, this equation can be found in (Oldham, 1962), and also in (Tu *et al.*, 2005).

Proof

Immediate from proposition 4. QED

Theorem 1 shows clearly that $r(X, (Y - X))$ is fully defined by the correlations $r(X, Y)$ and $r(X, X)$, along with the variability of Y. The correlation of X with itself, i.e. $r(X, X)$, is a prominent aspect of this equation, which drives its oddities. $r(X, X)$ reflects the coupling in the equation that arises because X appears in both the terms being correlated in $r(X, (Y - X))$. $r(X, X)$ is of course a constant, i.e. 1 for any X, so in fact, σ_Y and $r(X, Y)$, are the only variables; accordingly, their size determines the extent to which the imprint of X in $Y - X$ drives $r(X, (Y - X))$.

This leads to the key observation that, as σ_Y gets smaller, $r(X, (Y - X))$ tends towards $-r(X, X)$, which equals -1. In other words, as the variability of Y decreases, the imprint of X becomes increasingly prominent. This is shown in the next theorem.

Theorem 2

$$r\big(X, (Y - X)\big) \longrightarrow -r(X, X) = -1, \qquad \text{as } \sigma_Y \longrightarrow 0$$

Proof

The right hand side of equation *Imprint*, has five constituent terms, two in the numerator and three in the denominator. Of these five, three are products with the standard deviation of Y, i.e. σ_Y. Assuming all else is constant, as σ_Y reduces, the absolute value of each of these three terms reduces towards zero. The rate of reduction is different amongst the three, but they will all decrease. Accordingly, as σ_Y decreases, $r\big(X, (Y - X)\big)$ becomes increasingly determined by the two terms not

involving σ_Y, and thus, it tends towards $-\frac{r(X,X)}{\sqrt{+1}} = -r(X,X) = -1$.
QED

Equality of Residuals

An important finding of section 5 of the main text, is that the residuals resulting from regressing Y onto X are the same as regressing Y-X onto X. We show in this section, that this equality of residuals is necessarily the case.

We focus on the following two equations,

Eqn 1) $Y = \tilde{X}.\beta_1 + \varepsilon_1$

Eqn 2) $Y - X = \tilde{X}.\beta_2 + \varepsilon_2$

where $\tilde{X}$ is the $N \times 2$ matrix, with first column being X and second being the $N \times 1$ vector of ones (which provides the intercept term); β_1 and β_2 are 2×1 vectors of parameters and Y, X, ε_1 and ε_2 are $N \times 1$ vectors. As in the rest of this document, Y and X are our (demeaned) initial and outcome variables, while ε_1 and ε_2 are our residual error terms.

Proposition 9

If we assume that β_1 and β_2 are fit with ordinary least squares, with ε_1 and ε_2 the associated residuals, then, $\varepsilon_1 = \varepsilon_2$.

Proof

Under ordinary least squares, the parameters are set as follows.

$$\beta_1 = (\tilde{X}^T\tilde{X}\,)^{-1}\,\tilde{X}^T\,Y \qquad \text{(Eqn 3)}$$

$$\beta_2 = (\tilde{X}^T\tilde{X}\,)^{-1}\,\tilde{X}^T\,(Y - X) \qquad \text{(Eqn 4)}$$

We start with the second of these, and using left distributivity of matrices, and then substituting Eqn 3, we obtain the following.

$$\beta_2 = (\tilde{X}^T\tilde{X}\,)^{-1}\,\tilde{X}^T\,(Y - X) = (\tilde{X}^T\tilde{X}\,)^{-1}\,\tilde{X}^TY - (\tilde{X}^T\tilde{X}\,)^{-1}\,\tilde{X}^TX = \beta_1 - (\tilde{X}^T\tilde{X}\,)^{-1}\,\tilde{X}^TX$$

Using the fact that the variable X is demeaned, we can now evaluate the main term here as follows,

$$\beta_2 = \beta_1 - (\tilde{X}^T\tilde{X}\,)^{-1}\,\tilde{X}^TX = \beta_1 - \begin{pmatrix} X^2 & \Sigma X \\ \Sigma X & N \end{pmatrix}^{-1} \begin{pmatrix} X^2 \\ \Sigma X \end{pmatrix} = \beta_1 - \frac{1}{A}\begin{pmatrix} N & -\Sigma X \\ -\Sigma X & X^2 \end{pmatrix}\begin{pmatrix} X^2 \\ \Sigma X \end{pmatrix}$$

where X^2 is the dot product of X with itself, ΣX is the sum of the vector X, and $A = NX^2 - \Sigma X \Sigma X$ is the determinant of the matrix being inverted. From here we can derive the following,

$$\beta_2 = \beta_1 - \frac{1}{A}\begin{pmatrix} NX^2 - \Sigma X\Sigma X \\ -\Sigma X.X^2 + X^2.\Sigma X \end{pmatrix} = \beta_1 - \frac{1}{A}\begin{pmatrix} A \\ 0 \end{pmatrix} = \beta_1 - \begin{pmatrix} 1 \\ 0 \end{pmatrix}$$

We can then substitute this equality for β_2 in eqn 2 and re-arrange to obtain,

$$Y - X = \tilde{X}\beta_2 + \varepsilon_2 = \tilde{X}\left(\beta_1 - \begin{pmatrix} 1 \\ 0 \end{pmatrix}\right) + \varepsilon_2 = \tilde{X}\beta_1 - X + \varepsilon_2$$

It follows straightforwardly from here that,

$$Y - \tilde{X}\beta_1 = \varepsilon_2$$

i.e. $\varepsilon_1 = \varepsilon_2$, as required. QED

Proposition 9 shows that the residuals resulting from fitting equations 1 and 2 will be the same. A consequence of this is that the error variability will be the same. As a result of this, the factor that determines whether more variance is explained when regressing Y onto X or when regressing $Y - X$ onto X, is the variance available to explain. That is, the relative variance of Y and $Y - X$ drive the R^2 values of these two regressions. This then implicates the variance of Y and X and in fact their covariance (which impacts the variance of $Y - X$).

More precisely, we can state the following.

1) If $\sigma^2_{(Y-X)}$ is big relative to σ^2_Y, then regressing $Y - X$ onto X will explain more variability than regressing Y onto X.

2) If $\sigma^2_{(Y-X)}$ is small relative to σ^2_Y, then regressing $Y - X$ onto X will explain less variability than regressing Y onto X.

Supplementary Appendix B: illustrating the relationship between the correlations

```
% This function illustrates the relationship
function [r_XY,std_Y,r2,r3] = CheckEqn1()

noise = [0.01:0.01:1,2:100]; % controls r(X,Y)
scale = [0.01:0.01:1,2:100]; % controls sigma_Y/sigma_X
X = single(randn(1000,1));
for j=1:length(noise)
    Y = X + single(randn(1000,1).*noise(j)); %Y is X plus noise
    Y = zscore(Y); % then scale to X so the actual scaling is consistent
    for k=1:length(scale)
        Yl = Y.*scale(k); % rescale to control the variability ratio
        r_XY(j,k) = corr(X,Y); % calculate the correlation with outcomes

        r2(j,k) = corr(X,Yl-X); % calculate the correlation with change
        std_Y(j,k) = std(Yl)./std(X); % record the variability ratio
        r3(j,k) = eqn_r_X_XminusY(r_XY(j),std_Y(j,k)); % check Equation 1
    end
end

% display the resulting surface (Figure 1)
figure,surf(log(std_Y),r_XY,r3,'edgecolor','none')
lighting flat
l = light('Position',[50 100 100]);
l = light('Position',[50 100 -50]);
l = light('Position',[50 -100 -50]);
l = light('Position',[-50 -15 29]);
l = light('Position',[-50 -15 -29]);
l = light('Position',[-50 15 -29]);
l = light('Position',[50 15 -29]);
l = light('Position',[50 15 -50]);
shading interp
xlabel('log ( sigmaY / sigmaX )')
ylabel('r(X,Y)')
zlabel('r(X,Y-X)')

% confirm that equation 1 does actually match 'empirical' r(X,Y-X)
figure,scatter(r2(:),r3(:))
xlabel('Empirical coefficients')
ylabel('Derived coefficients')

end

% This function implements Equation 1
function res = eqn_r_X_XminusY(r_XY,std_Y)

res = (((r_XY.*std_Y) - 1) ./ sqrt(1 + (std_Y).^2 - (2*(r_XY.*std_Y))));

end
```
